# The role of PPARγ in childhood obesity-induced fractures

**DOI:** 10.1186/s12263-019-0653-7

**Published:** 2019-11-27

**Authors:** Matthew R. McCann, Anusha Ratneswaran

**Affiliations:** 1 Sydney Medical School, University of Sydney, Sydney, NSW 2006 Australia; 2 Western Bone & Joint Institute, University of Western Ontario, London, ON N6A 2J9 Canada; 3 Department of Physiology and Pharmacology, University of Western Ontario, London, ON N6A 2J9 Canada; 4 Department of Genetics and Development, Krembil Research Institute, University Health Network, Toronto, ON M5T 0S8 Canada

**Keywords:** Bone, Bone mineral density, Childhood obesity, Osteoporosis, Bone fractures, Slipped capital femoral epiphysis, Blount’s disease, PPARγ, Osteoblast, Osteoclast

## Abstract

Globally, obesity is on the rise with ~ 30% of the world’s population now obese, and childhood obesity is following similar trends. Childhood obesity has been associated with numerous chronic conditions, including musculoskeletal disorders. This review highlights the effects of childhood adiposity on bone density by way of analyzing clinical studies and further describing two severe skeletal conditions, slipped capital femoral epiphysis and Blount’s disease. The latter half of this review discusses bone remodeling and cell types that mediate bone growth and strength, including key growth factors and transcription factors that help orchestrate this complex pathology. In particular, the transcriptional factor peroxisome proliferator-activated receptor gamma (PPARγ) is examined as it is a master regulator of adipocyte differentiation in mesenchymal stem cells (MSCs) that can also influence osteoblast populations. Obese individuals are known to have higher levels of PPARγ expression which contributes to their increased adipocyte numbers and decreased bone density. Modulating PPAR*gamma* signaling can have significant effects on adipogenesis, thereby directing MSCs down the osteoblastogenesis pathway and in turn increasing bone mineral density. Lastly, we explore the potential of PPARγ as a druggable target to decrease adiposity, increase bone density, and be a treatment for children with obesity-induced bone fractures.

## Background

### Childhood obesity and bone density

Obesity has become an international health risk, with reports indicating its status has been elevated to that of a global pandemic [1]. Assessment of the *2013 Global Burden of Disease* showed that the percentage of individuals with a body mass index (BMI; weight in kg/height in m^2^) greater than 25 has increased from 28.8 to 36.9% in men and from 29.8 to 38.0% in women between 1980 and 2013 [2]. Rates of childhood and adolescent obesity are also increasing in both developed and developing countries, with no national initiatives successfully decreasing obesity rated in the past 30 years [2]. The cost of obesity and its associated comorbidities are staggering, and in 2014, it was estimated that the total global cost of obesity was $4 trillion (USD) [3].

In adults, being overweight is defined as having a BMI greater than 25 and obesity is defined as a BMI greater than 30 [4]. BMI is still an accurate and valid method of determining adiposity in children and adolescents [5]. However, unlike adults, there are no set numerical values, because as children grow, their body composition and bone structure are altered rapidly. As such, obesity is defined as having a BMI greater than the 95th percentile for age and sex. Childhood obesity has been associated with numerous chronic diseases including type 2 diabetes mellitus, cancer, hypertension, hypercholesterolemia, and cardiovascular and liver diseases [6]. However, the effect obesity has on the musculoskeletal system is less established [7].

## Main text

### Bone remodeling and adaptation

Bone is highly responsive to its environment. Wolff’s law demonstrates that bone is able to adapt and provide adequate strength and rigidity to sustain the mechanical and physiologic functions of the body. Bone is able to achieve these properties by sensing repeated loading or nutrient availability, which in turn leads to regulation of the balance between bone deposition and resorption [8]. This active process of bone turnover in children is termed modeling, and in adults, it is called remodeling. In healthy individuals, the balance shifts throughout life, with early years favoring selective deposition, while in the middle of life, resorption and deposition are balanced and stable. Lastly, in the fifth decade of life, there is a shift to increased bone resorption and decreased bone density. Bone is able to regulate this turnover process through two major cell types: the mesenchymal-derived osteoblasts that deposit new bone and osteoclasts from the hematopoietic lineage that resorb bone.

In early life, bone modeling is critical because bone mineral density (BMD) is mostly accrued during adolescence and peak bone density is thought to occur between the ages of 20 and 30 [9]. It is critical to achieve a high peak bone mass for proper skeletal function but also to avoid potential issues with low BMD later in life [10]. Osteoporosis is characterized by decreased BMD and marked destruction of the trabecular bone struts in spongy bone. This often results in increased bone frailty and greater likelihood of fracture. While postmenopausal osteoporosis is a leading cause of fracture in women, men also have decreases in BMD as they age but this is thought to be due to decreases in circulating testosterone levels, higher fat mass, and decreased muscle mass [11]. The clinical definition for adult osteoporosis is a BMD that is 2.5 standard deviations or more below the population mean of a healthy 30-year-old adult [12].

While age- and drug-induced osteoporoses are well known, juvenile osteoporosis is rare and less defined, and its etiology is not completely known [13]. Juvenile osteoporosis develops prepubertally and often leads to compression fractures in the vertebrae and the metaphyses of long bones [14]. The specific location of these fractures indicates that it affects the trabecular bone in a similar manner to adult osteoporosis. These children have BMD that is less than 2.5 standard deviations from aged-matched healthy controls; however, a diagnosis of juvenile osteoporosis cannot be made on dual-energy X-ray absorptiometry (DXA) results alone, and multiple criteria including past fracture history and frequency, diet, ethnicity, and height and weight must be assessed [15].

### Clinical studies of adiposity-induced low BMD

Adults with a high BMI are at a lower risk for osteoporosis, as increased weight positively correlates with increased BMD and lower risk of fractures [16]. While there have been are conflicting reports [17], the consensus is that children with obesity have lower BMD and increased fracture rates when compared to normal weight children [18].

Compelling evidence was the large-scale chart review cross-sectional study that evaluated 913,178 patients between the ages of 2 to 19 years [19]. In this study, BMI was stratified into five weight classes (underweight, normal weight, overweight, moderate obesity, and extreme obesity), and records were screened for lower extremity fractures. The overweight, moderate, and severely obese all had increased odds of fracture in the foot, ankle, knee, and tibia/fibula when compared to normal weight controls after adjustment for sex, race, age, neighborhood education, and medical care benefit use. Notably, the increased fracture risk was higher in those patients who had a higher BMI. A more recent cross-sectional study of 2213 children found that only overweight children had a higher risk lower limb fractures, and there was no association between obese patients and normal controls [20]. Further assessment of upper limb fractures demonstrated that children with forearm fractures were more likely to be higher BMIs when compared to the age and region reference population [21]. Interestingly, obesity only appears to affect incidences of fractures in children and does not appear to affect the severity of fractures [17].

There are many potential explanations why children with obesity are at a higher risk for fractures, including altered gait and poor balance, which results in increased susceptibility to falls [22]. Finite element modeling of the pelvic bone showed that increased weight and therefore higher impact forces in children with obesity further exacerbated fracture risk in this population [23]. Lifestyle factors also contribute heavily to obesity in children, including increased sedentary behavior, poor diet [24], and poor sleeping habits which can lead to weight gain [25]. Lastly, excessive adipose tissue itself can have direct molecular and hormonal effects on bone density during this critical period of rapid skeletal growth [26].

The importance of adiposity in bone development and modeling cannot be understated, as children with inadequate levels of fat deposits fail to begin skeletal maturation during puberty [27]. Conversely, excess adiposity has shown to increase bone diameter, yet these bones are less structurally sound and have a higher incidence of fracture. There is current debate and conflicting studies as to whether increased adiposity leads to larger bones, increased or decreased density, or increased fractures rates (Table 1). Not only are obese children more likely to have fractures but once they do, these children have a higher rate of improper bone reductions and require more subsequent manipulations to correct the misaligned bones [49]. Additionally, children with higher BMIs have a greater prevalence of open reduction surgery to repair their factures [49], which leads to increased surgery complications [50] and more cosmetic scarring compared to closed reductions. Fueling further complexity, it has also been shown that where the deposition of adiposity is localized also has an impact on bone strength. When adiposity is deposited as visceral fat, it leads to decreased bone density in the vertebral bones [42] or femur [39]. Yet, adiposity deposition in the subcutaneous fat has positive associations with bone structure and strength [39]. In healthy adult women, lean mass was shown to have a positive correlation to BMD, while fat mass demonstrated a negative correlation, with the threshold for fat mass being in the 30 to 38% body fat range [51]. The threshold for where body fat percentage becomes detrimental in the pediatric population is not known and warrants further study.

**Table 1:** Summary of pediatric studies evaluating adiposity and bone mineral density and fractures

Study	Study design	Population	Age (years)	Gender	Geographical location	Obesity assessment	Bone/fracture assessment	Results
Goulding et al. [28]	Case-control	206 (3 with distal forearm fracture)	3–15	Female	Dunedin, New Zealand	BMI Total fat mass	DXA: radius DXA: lumbar DXA: whole body Past medical records	Girls with forearm fractures have lower BMD and higher adiposity than non-fracture controls
Goulding et al. [29]	Cohort study	200 (100 forearm fracture)	10 ± 2.9	Female	Dunedin, New Zealand	BMI	DXA: radius DXA: lumbar DXA: whole body Past medical records	Girls with higher BMI and lower bone density were at greater risk for fracture
Goulding et al. [30]	Case-control	200 (100 forearm fracture)	3–19	Male	Dunedin, New Zealand	BMI Total fat mass	DXA: radius DXA: hip DXA: lumbar DXA: whole body Past medical records	Boys with forearm fracture were more overweight and had lower radial BMD
Skaggs et al. [31]	Case-control	100 (50 with forearm fracture	4–15	Female	Los Angeles, California, USA	BMI	CT: radius Past fracture history	Girls with forearm fractures had a smaller radius and higher weight compared to non-fracture controls
Davidson et al. [32]	Case-control	50 (25 obese)	4–17	Male	Dunedin, New Zealand	BMI	DXA: radius DXA: whole body	Obese children were at greater risk of forearm fracture
Goulding et al. [33]	Cross-sectional	90 children with forearm fracture	5–19	Male and female	Dunedin, New Zealand	BMI	DXA: lumbar DXA: hip DXA: forearm DXA: whole body Past fracture history	Children with repeated forearm fractures had lower radial BMD and higher BMI
Taylor et al. [34]	Retrospective cross-sectional	355 (227 overweight)	12.2 ± 2.8	Male and female	Washington, DC, USA	BMI	DXA: lower extremities Past fracture history	Overweight children had a greater prevalence of fracture
Janicka et al. [35]	Cross-sectional	300 healthy cases	13–21	Male and female	Los Angles, California, USA	BMI	CT: femur CT: lumbar DXA: lumbar	Total body fat mass was not associated with BMD or cortical bone structure in males. Females had a negative association between DXA leg BMD and fat mass
Pollack et al. [36]	Cross-sectional	115	18.2 ± 0.4	Male and female	Athens, Georgia, USA	BMI	DXA: whole body pQCT: radius pQCT: tibia	Body fat percentage was inversely correlated with cortical bone size and strength indices
Wetzsteon et al. [37]	Longitudinal	445 (143 obese)	9–11	Male and female	British Columbia, Canada	BMI	DXA: whole body pQCT: tibia	In overweight children, bone strength adapted to greater lean mass but did not respond to excess fat mass
Dimitri et al. [38]	Cross-sectional	103 children (52 obese)	11.7 ± 2.8	Male and female	Sheffield, UK	BMI Total fat mass	DXA: lumbar DXA: radius DXA: whole body Past fracture history	Obese children with prior fracture had reduced BMD
Gilsanz et al. [39]	Cross-sectional	100 healthy adolescents and young adults	15–25	Female	Los Angeles, California, USA	BMI Waist circumference	CT: waist CT: femur	High levels of visceral fat were associated with decreased femoral cortical and cross-sectional area. Subcutaneous fat had beneficial effects in these measurements.
Farr et al. [40]	Cross-sectional	198 healthy children	8–15	Male and female	Minnesota, USA	Total body fat mass	DXA: whole body HRpQCT: radius HRpQCT: tibia	Total body fat mass affected the distal tibial failure and no effect on radius.
Sayers et al. [41]	Longitudinal cohort	3914	Avg.: 13.8	Male and female	Southwest England	Total body fat and lean mass	DXA: total hip DXA: femoral neck	In females there was a positive relationship between adiposity and femoral neck buckling
Russell et al. [42]	Cross-sectional	30 (15 obese, 15 normal weight)	12–18	Female	Boston, Massachusetts, USA	BMI	MRI: lumbar DXA: lumbar DXA: hip DXA: whole body	Visceral adipose levels inversely correlated with vertebral bone density in females
Wey et al. [43]	Cross-sectional and longitudinal	370	8–18	Male and female	South Dakota, USA		DXA: whole body pQCT: radius	Higher fat mass was associated with reduced bone size. Longitudinal gain of fat negatively impacted cortical area.
Kessler et al. [19]	Cross-sectional	913,718	2–19	Male and female	California, USA	BMI	Past fracture history	Higher BMI was associated with increased risk of lower extremity fractures
Fornari et al. [44]	Retrospective cross-sectional	922 fracture cases	5.0 ± 2.5	Male and female	California, USA	BMI	Past fracture history	Children with obesity were at a greater risk of and severity for lateral condyle factures.
Laddu et al. [45]	Longitudinal	260 healthy children	8–13	Female	Arizona, USA	BMI	DXA: whole body pQCT: femur pQCT: tibia	At baseline, visceral fat mass was a positive predictor of bone strength. Longitudinally, central fat mass may hinder cortical bone strength.
Sabhaney et al. [20]	Cross-sectional	2213 (1078 had fracture, 316 obese)	9.5 ± 4.2	Male and female	British Columbia and Ontario, Canada	BMI	Past fracture history	Obese children had a minor decreased odds of fracture relative to normal weight children
Kwan et al. [17]	Retrospective cross-sectional	1340 patients with extremity factures	2–17	Male and female	Toronto, Ontario, Canada	Weight-for-age > 95th percentage	Past fracture history	Obese children were not at an increased risk of sustaining more severe extremity fractures or subsequent complications then non-obese children.
Gilbert et al. [46]	Retrospective chart review	331 femur and tibia factures	2–14	Male and female	Alabama and Tennessee, USA	BMI	Past fracture history	Obese patients were twice as likely to have fractures involving the physis.
Moon et al. [21]	Cross-sectional	401 acute upper limb fracture	3–18	Male and female	Southhampton, UK	BMI SFT: triceps SFT: subscapular	Upper limb fractures in the previous 60 days	Overweight and obese prevalence was higher in children with forearm and upper arm fractures. More pronounced in boys upper limb fractures
Manning et al. [47]	Retrospective case-control	929 forearm fractures	0–17 years	Male and female	Washington, DC, USA	Weight-for-age/sex > 95th percentage	Past radial bone fractures	Children with weight greater than the 95th percentile of age/sex had higher odds of ground-level fractures.
Khadilkar et al. [48]	Cross-sectional	245	6–17	Male and female	Pune, India	BMI	DXA: whole body	Total BMC, BMC, and bone area are lower in increasing BMI

The reason underlying differential effects on BMD from adiposity location is unknown. It is believed that weight gain in adolescence may limit the periosteum (outer layer of bone) that normally expands during this period of rapid growth, thereby decreasing the bones structure and strength relative to the increasing body weight [52]. Further, there is little evidence exploring if the rate of weight gain affects BMD both acutely and chronically. The rate could have varying effects depending on which stage of puberty it occurs or if it transcends the years of puberty.

### Slipped capital femoral epiphysis

One of the most well-known effects of obesity on bone is the increased prevalence of slipped capital femoral epiphysis (SCFE) [7]. This condition is the result of a non-traumatic fracture between the proximal femoral epiphysis and metaphysis that typically occurs during adolescent growth. The exact cause of SCFE is unknown, but it is multifactorial disease in which obesity is thought to be a key contributor, and that increased weight bearing alters loading to the hip joint [7]. A Scottish study reported that the incidence of SCFE rose from 2.78 per 100,000 children in 1981 to 9.66 per 100,000 in 2000 [53]. This was a 2.5-fold increase in two decades, which parallels childhood obesity rates. A similar trend was also noted in New Mexico, USA, with a 3.4-fold increase in 2006 compared to the 1960s [54]. It is estimated that 30–50% of children with SCFE are overweight [55]. The first correlation between high BMI and increased rate of SCFE was in 2003 by Poussa et al. [56]. Since then, there have been other studies showing similar results [57, 58] and that the severity of SCFE increases as BMI increases [59], while the incidence of bilaterally SCFE also increases [60]. There are conflicting reports if SCFE is related to vitamin D intake [61], and yet, a new study shows an association between elevated serum leptin levels and SCFE, regardless of BMI [62]. This further demonstrates that SCFE is multifactorial disease that may not be strictly dependent on the altered biomechanics hypothesis. SCFE patients with an increased BMI had a worse 20-year follow-up [63], but reduction of BMI to lower than the 95th percentile post-surgery decreased the odds of bilateral SCFE development by 84% [64].

### Blount’s disease

Another bone deformity associated with childhood obesity is Blount’s disease, also known as tibia vara. This is a progressive disorder that results from altered growth of the proximal tibia physis and results in varus deformation of the tibia including tibial rotation and procurvatum (backwards bending) [65]. While this disease is relatively rare, two thirds of children with this condition are obese [66], and the rates of Blount’s disease are rising in parallel to the increasing prevalence of obesity in children [67]. An 8-year longitudinal study showed that patients with early-onset Blount’s disease (< 4 years of age) have a greater severity of the disease [68], which can also lead to early degenerative osteoarthritis in early adulthood [69].

The current etiology of Blount’s disease is unknown. In addition to obesity, there appear to be differences between age, sex, and race [65] and minerals such as zinc and copper [70], and there are mixed results regarding association with vitamin D levels [71, 72]. In these early-onset cases, excess weight causes bowing of the tibia, leading to altered pressure on the epiphysis, improper ossification of the cartilage in the medial metaphysis, and insufficient growth of the medial physis [73]. The longitudinal growth of the tibia via the physis is disrupted by these compressive forces in a process called the Hueter-Volkmann’s law [74]. While weight may be a contributing factor to alterations in the growth plate, it is unlikely that this is the sole cause for the development of Blount’s disease [75]. Histopathological evaluation of the growth plate in these patients demonstrated cellular disorganization of the growth plate and impaired differentiation of chondrocytes into hypertrophic cells (a similar cellular disorganization is seen in SCFE) [76]. It is thought that PPARγ initially causes delayed maturation in the growth plate and the added mechanical stress from the increased body weight results in impaired terminal differentiation and malalignment [77].

Upon clinical diagnosis of Blount’s disease, surgical intervention is usually recommended in conjuncture with a pediatric obesity specialist to implement a weight loss program to prevent reoccurrence [78]. However, after successful surgical intervention, correction of the misalignment, and post-surgical nutritional counseling, 78% of these patients still continued to gain weight after 48 months of follow-up [79].

In summary, it is apparent obesity can have devastating effects on the skeletal system, and these conditions cannot be attributed to the effects of the increased weight bearing alone. Therefore, there are potential other mediators that may be driving decreased BMD in children with obesity. Some of these mediators may stem from factors involved in bone development, such as the opposing lineage differentiation pathways between adipose tissue and bone tissue. To further understand the complex relationship between childhood obesity, decreased BMD, and conditions like SCFE and Blount’s disease, it is critical to examine which molecular regulators are responsible for the regulation of bone turnover and adiposity and how they change in the associated pathologies.

### Cellular and molecular regulators of bone remodeling

Bone is under strict control of its remodeling, through a delicate and complicated mechanism known as bone coupling which regulates bone turnover of the whole skeleton to approximately 10% per year [80]. In this process, both trabecular and cortical bone are degraded and rebuilt, yet at different rates. The initiation step is the recruitment of hematopoietic precursor cells through capillary blood vessels that supply the cortical bone or precursors that are already present in the marrow cavity for trabecular bone. Homing of these precursor cells is directed by endocrine and paracrine factors released from endothelial cells, such as nitric oxide, vascular endothelial growth factor (VEGF), macrophage colony-stimulating factor (M-CSF), and a receptor activator of nuclear factor-κB ligand (RANKL) [73]. The latter two factors are secreted from osteoblasts and their mesenchymal precursors to regulate osteoclast recruitment and therefore bone resorption. Additionally, osteoblasts have the ability to secrete osteoprotegerin, which can bind and sequester RANKL, thereby inhibiting its ability to bind to RANK and thus limiting osteoclast differentiation [81].

**Fig. 1:**
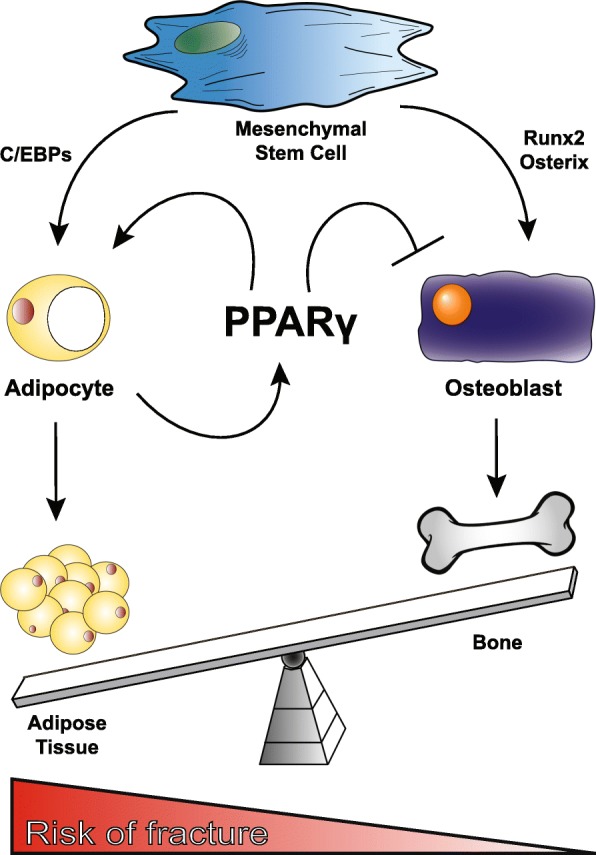
Effects of PPARγ activation on adipocyte and osteoblasts differentiation. A simplified schematic diagram that shows the effects of PPARγ on mesenchymal stem cell differentiation. PPARγ activation, in conjunction with C/EBPs, is able to cause differentiation of mesenchymal stem cells down the adipogenesis pathway and thereby inhibiting differentiation of osteoblasts. PPARγ activation results in a positive feedback loop that increases adipogenesis. As a result, more adipose tissue is accumulated at the expense of osteoblastogenesis and matrix deposition. This switch inhibits bone mineral density and bone functional loading capacity and therefore leads to an overall increased risk of fracture in the childhood population

Importantly, osteoblasts have their origins from a separate lineage than the osteoclast, as they are derived from MSCs that are situated in the bone marrow itself. These MSCs are multipotent and are able to differentiate into multiple cell types including, cartilage, tendons, myocytes, and notably osteoblasts and adipocytes [82]. Each of these committed differentiation pathways has their own lineage commitment and maturation factors. These factors can be either exogenous to the cell such as hormones, growth factors, and physical environment (stiffness of the extracellular matrix) or endogenous mechanisms such as age and metabolism. The differentiation of MSCs into adipocytes is regulated by PPARγ and CCAAT-enhancer-binding proteins (C/EBPs) [83] and osteoblast differentiation is governed by Runx2 and Osterix [84] (Fig. 1).

### Role of PPARγ in osteoblastogenesis and bone density

PPARγ is a nuclear receptor that is primarily expressed in adipose tissue and in MSCs residing in the bone marrow [85]. PPARγ is a master regulator of adipogenesis [86], indicating that it is at the top of the regulatory network hierarchy and is able to govern lineage specification. It has been described as a “metabolic switch” for stem cell fate in the mesenchymal and hematopoietic lineages [87].

PPARγ’s role as a master regulator was shown in a well-designed study by Akune et al., where genetically modified embryonic stem cells lacking PPARγ spontaneously differentiated into osteoblasts and failed to produce adipocytes [85]. They further conducted an in vivo mouse model experiment which showed that PPARγ haploinsufficient mice had increased bone mass and osteoblastogenesis from bone marrow progenitors, indicating that PPARγ is indeed a suppressor of MSC to osteoblast lineage differentiation. However, it was unknown if these effects were acting directly on osteoblasts or indirectly through osteoclasts. Follow-up studies were conducted using selective deletion of PPARγ from the hematopoietic lineages and thus subsequent inactivation in osteoclasts while maintaining expression in osteoblasts [88]. In these conditions, the increased bone mass and reduced marrow cavity space are a consequence of impaired osteoclast differentiation and thus impaired bone resorption. Therefore, PPARγ can elicit its response through both promoting osteoclast-driven bone resorption and/or by decreasing osteogenesis by inhibiting MSC to osteoblast differentiation. Compelling in vivo data has shown that mutating a phosphorylation site of PPARγ and thus rendering its inhibition properties inactive results in unregulated PPARγ activity that decreased bone volume in trabecular bone compared to wild-type littermates. This study also demonstrated that in these mutant mice, adipocytes markers were elevated and isolated bone marrow stem cells had increased levels PPARγ and preferentially differentiated to adipocytes rather than osteoblasts [89]. It was also observed that phosphorylated Runx2 (a driver of osteoblastogenesis) and osteoblastogenesis were inhibited. Overall, this indicates that in the bone marrow, Runx2 and PPARγ are reciprocally controlled and are important regulators of bone formation and turnover.

Clinically, screening obese patients for PPARγ expression indicated that levels of PPARγ increased in proportion to increased BMI [90]. Additionally, adipocyte differentiation was found to perpetuate through multiple positive feedback loops, which seek to drive adipogenesis [91], making it difficult to break the cycle of adipogenesis once it has begun. Pharmacological activation of PPARγ can be achieved by the administration of antidiabetic drugs, the thiazolidinediones, in which rosiglitazone is prime example. These drugs regulate adipocytes to produce endocrine factors that make peripheral tissue more sensitive to insulin, yet they also increase fat storage [92]. In a 14-week randomized, double-blind, placebo-controlled administration of the rosiglitazone on post-menopausal women inhibited bone formation and decreased BMD as early as 4 weeks into the treatment and was sustained for the rest of the trial [93]. This suggests that promotion of PPARγ activity through pharmacological agonist rosiglitazone has opposite effects to genetic inhibition of PPARγ, and they may act through the same mechanisms, as expected.

PPARγ’s effects on bone homeostasis are known to be context specific [87]. In an animal model, rosiglitazone was shown to have age-dependent effects; in young mice, rosiglitazone decreased the rate bone formation, while in old mice, there was increased bone loss [94]. This has been shown to be due to downregulating expression of *Runx2*, *Osterix*, and *Opg* [95, 96]. This distinction indicates that there must be endogenous changes present within the bone marrow milieu. Aging has been shown to increase the expression of PPARγ [97] and decreases its interaction with its coactivator SRC-1, leaving it primed for adipogenesis later in life [98]. These age-specific changes in PPARγ resemble how adiposity affects bone density and fracture risk differently in children and adults. This is further demonstrated between two randomized clinical trials testing the effect of rosiglitazone on maintaining long-term glycemic control. The adult-based ADOPT (A Diabetes Outcome Progression Trial) found that women receiving rosiglitazone alone had a greater propensity of upper and lower limb fractures then either metformin or glyburide alone [99]. Conversely, the child Treatment Options for type 2 Diabetes on Adolescent and Youth (TODAY) found no differences in bone mineral content or fracture rate between metformin alone, metformin plus rosiglitazone, or metformin plus lifestyle intervention [100]. Although, these results should be taken with caution as the TODAY trial had low sample size. A follow-up report on the TODAY trial showed that patients who were given metformin and rosiglitazone had a lower BMD compared to patients of in metformin and lifestyle moderation (200–300 min/week of physical activity and improved diet) arm, after 24 months [101]. Direct comparison is complicated as there was no rosiglitazone alone treatment in the TODAY trial and that the lifestyle intervention could affect bone accrual.

## Conclusion

Childhood obesity has significant effects on the musculoskeletal system and in particular bone density and fracture rate. Therefore, it is important to know how obesity affects the skeleton during adolescence, as this is a critical window of bone growth and structural support. A key mediator in this process is PPARγ as it directly effects adipogenesis and indirectly alters osteoblastogenesis. SCFE and Blount’s disease are two serious bone conditions associated with childhood obesity. To our knowledge, there are no preclinical models of SCFE or Blount’s disease, but evidence from adult trials of PPARγ agonist rosiglitazone indirectly suggest that agonism of the PPARγ pathway results in decreased BMD and increased fracture risk. However, preclinical evidence suggests that this effect may not be as severe before adulthood. Future studies should determine if PPARγ is a suitable candidate for pharmacological intervention to treat both obesity and childhood low bone density which may influence the incidence of SCFE and Blount’s disease.

## Data Availability

Not applicable

## Abbreviations

ADOPT: A Diabetes Outcome Progression Trial
BMD: Bone mineral density
BMI: Body mass index
C/EBPs: CCAAT-enhancer-binding proteins
DXA: Dual-energy X-ray absorptiometry
HRpQCT: High-resolution peripheral quantitative computed tomography
MSCs: Mesenchymal stem cell
PPARγ: Peroxisome proliferator-activated receptor gamma
pQCT: Peripheral quantitative computed tomography
SCFE: Slipped capital femoral epiphysis
SFT: Skinfold thickness
TODAY: Treatment Options for type 2 Diabetes in Adolescents and Youth
